# Xanthones of Lichen Source: A 2016 Update

**DOI:** 10.3390/molecules21030294

**Published:** 2016-03-02

**Authors:** Pierre Le Pogam, Joël Boustie

**Affiliations:** Laboratoire de Pharmacognosie, Equipe PNSCM, (ISCR UMR CNRS 6226), Faculté des Sciences Pharmaceutiques et Biologiques, 2 Avenue du Professeur Léon Bernard, 35043, Rennes Cédex, France; pierre.lepogam-alluard@univ-rennes1.fr

**Keywords:** biosynthesis, polyketides, fungi, NMR spectroscopy, bioactivity, lichexanthone, islandicin, thiomelin, secalonic acids

## Abstract

An update of xanthones encountered in lichens is proposed as more than 20 new xanthones have been described since the publication of the compendium of lichen metabolites by Huneck and Yoshimura in 1996. The last decades witnessed major advances regarding the elucidation of biosynthetic schemes leading to these fascinating compounds, accounting for the unique substitution patterns of a very vast majority of lichen xanthones. Besides a comprehensive analysis of the structures of xanthones described in lichens, their bioactivities and the emerging analytical strategies used to pinpoint them within lichens are presented here together with physico-chemical properties (including NMR data) as reported since 1996.

## 1. Introduction

Xanthones are ubiquitous polyphenolic compounds displaying a common 9*H*-xanthen-9-one scaffold [1]. Bioactivities of these compounds depend on their tricyclic core as well as on the nature and/or position of their highly diverse substituents, making them a “privileged structure” [2] likely to bind a variety of targets [3]. Thus, more than 250 of them were shown to display significant bioactivities including antimicrobial, antioxidant and cytotoxic activities [4]. Even though xanthones might be regarded as non-specific lichen compounds compared to other structural classes (e.g., depsides, depsidones, dibenzofurans…), the specific biosynthetic pathway of fungal xanthones results in novel substitution patterns, highlighting the interest of lichen xanthones. Furthermore, since the compendium of lichen substances established by Huneck and Yoshimura in 1996 [5], various xanthones of unexpected structures were isolated from lichen sources, further strengthening the interest in the chemodiversity of this source. This review aims at gathering all the recent data regarding the lichen xanthones that has appeared since 1996. A brief insight is given into the biosynthetic schemes of lichen xanthones with a focus on elements accounting for their specific substitution. Subsequently, the structures of all lichen xanthones isolated since 1996 are given and their available physico-chemical properties, including NMR data, are further compiled alongside their published bioactivities.

## 2. Current Data Regarding the Biosynthesis of Xanthones within Lichens

As with most lichen metabolites [6], the biosynthesis of xanthones proceeds through the polyacetate/polymalonate pathway, featuring the internal cyclization of a single folded polyketide chain. Two distinct series of xanthones are obtained, depending on this folding pattern.

**Figure 1:**
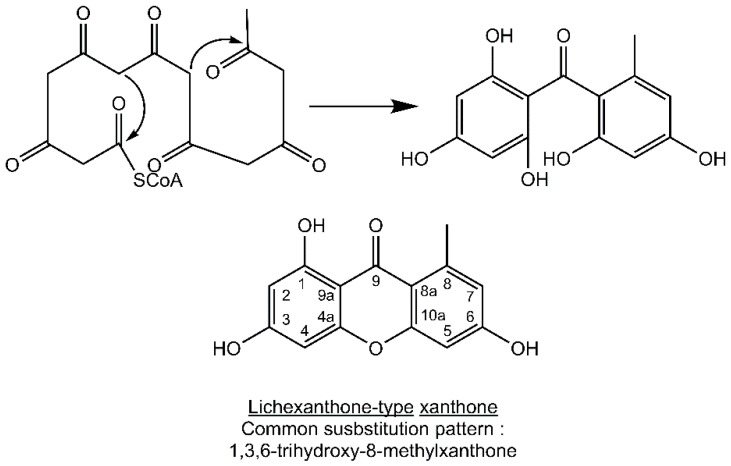
Proposed biosynthetic pathway for lichexanthone-type lichen xanthones. Adapted from [7].

Most lichen xanthones arise through the folding of a polyketide intermediate as described by Cacho *et al.* [7], resulting in structures displaying a methyl group in position 8 (Figure 1). Aldol condensation and Claisen-type cyclization and release and yield a benzophenone intermediate that might spontaneously dehydrate to obtain the central pyrone core. This biosynthetic scheme gives rise to the common oxygen substitution pattern of lichexanthone and norlichexanthone (1,3,6-trihydroxy-8-methylxanthone).

**Figure 2:**
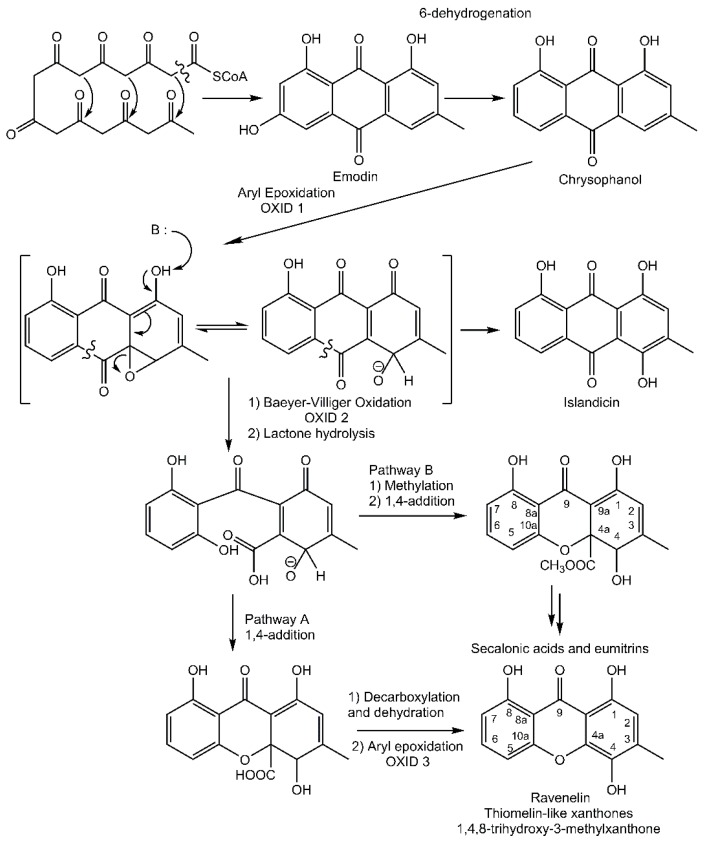
Proposed biosynthetic pathway for thiomelin-type lichen xanthones. Adapted from [12].

In contrast, a limited number of structures arise via a distinct biosynthetic pathway that leads to the ravenelin skeleton, with the methyl group in position 3. This biosynthetic scheme begins with the widespread anthraquinone emodin as a precursor [8] (Figure 2). To begin with, the hydroxyl group on C-6 of the emodin disappears (yielding chrysophanol) as it was observed in cell-free preparations of the fungus *Pyrenochaeta terrestris* [9,10]. The hydroxyl group on C-4 is then incorporated after the oxidative ring opens [11]. Deeper insights into this latter biosynthetic event were discussed by Henry and Townsend [12], who proposed an aryl epoxidation across an A-ring edge of chrysophanol to yield an intermediate that lost its A-ring aromaticity. Under this scheme, this intermediate, stabilized by a hydrogen bond between its newly formed phenol group and the neighboring quinone group, recovers its A-ring aromaticity to grant islandicin as a shunt product. An alternative for this intermediate is to undergo a second oxidation, most likely by the same P450 oxygenase, to afford a Baeyer-Villiger cleavage of the central quinone ring to yield an *ortho* carboxybenzophenone that might follow several metabolic fates. A first possibility is the 1,4-addition of a B-ring phenol to the A-ring dienone followed by dehydration and decarboxylation to access ravenelin-like xanthones after a final oxidation [12]. This results in xanthones displaying an archetypical 1,4,8-trihydroxy-3-methylxanthone skeleton. A second metabolic pathway, granting access to eumitrins and secalonic acids, is assumed to include a methylation of the carboxy group to prevent its subsequent elimination after a similar 1,4-addition. Finally, a subsequent 1,2-addition to the benzophenone intermediate leads to further cores similar to that of tajixanthone produced by *Aspergillus variecolor*, a skeleton thus far unknown from lichens.

**Figure 3:**
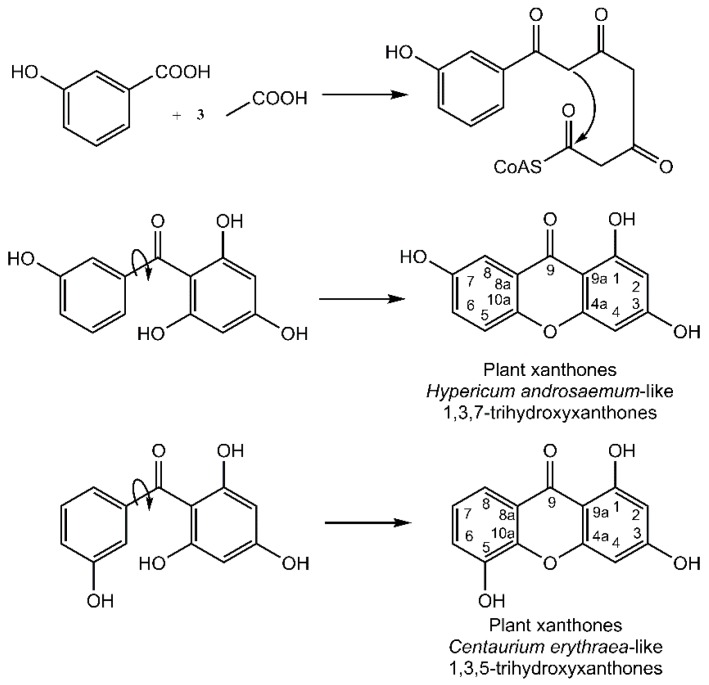
Biosynthesis of xanthones within plants.

It is noteworthy that the xanthone nucleus of plants is of mixed biosynthetic origin with the A-ring being acetate-derived whereas shikimic acid pathway–derived 3-hydroxybenzoic acid gives rise to the C-ring (Figure 3). Aromatization of the side chain leads to a freely rotating benzophenone intermediate that can further cyclize to yield the xanthone core. Regioselective oxidative coupling can then yield a 1,3,7-trihydroxyxanthone (*Hypericum androsaemum*) or 1,3,5-trihydroxyxanthone (*Centaurium erythraea*) [4].

Given these different biosynthetic pathways, only few lichen xanthones are known from non-lichenized organisms. However, some can be produced by higher plants, such as lichexanthone (e.g., *Anthocleista djalonensis* [13], *Croton cuneatus* [14]*, Cupania cinerea* [15]*, Feroniella lucida* [16]*, Minquartia guianensis* [17], *Zanthoxylum microcarpum* [18], *Z. valens* [18]), vinetorin (*Hypericum ascyron* [19]). Likewise, non-lichenized fungi have the ability to synthesize some lichen xanthones, such as lichexanthone from various *Penicillium* [20], norlichexanthone from *Penicillium patulum* [21] and the endolichenic fungus *Ulocladium* [22]; 1,3,6-trihydroxy-8-methylxanthone (also known as griseoxanthone C) is a precursor of aflatoxins—a group of significant environmental mycotoxins—first reported from *Penicillium patulum* [23]. Secalonic acids are also mycotoxins produced by a wide array of fungi [24].

Likewise, very few xanthones are common to higher plants and fungi, with some such examples being 1,7-dihydroxyxanthone (known from the plants *Vismia parviflora* [25] and *Weddelina squamulosa* [26] while also being produced by a *Penicillium* strain [27]) 1,8-dihydroxy-3-methoxy-6-methylxanthone (plant *Cassia obtusifolia* [28] and fungus *Astrocystis* sp. BCC 22166 [29]), pinselin (plant *Cassia occidentalis* [30] and several fungal strains including an endophytic *Phomopsis* sp. [31], *Talaromyces bacillosporus* [32] and the marine-derived *Engyodontium album* [33]), 6-*O*-methyl-2-deprenylrheediaxanthone B (plant *Garcinia vieillardii* [34] and fungus *Phomopsis* sp. [31]), as well as 8-desoxygartanin (produced by both *Garcinia mangostana* and *Streptomyces rishiriensis* 265-P5921 (according to the Dictionary of Natural Products)). It can therefore be stated that xanthones are highly unique to each realm, legitimating joint efforts on higher plants, non-lichenized fungi and lichens to widen the chemical diversity of these privileged structures.

**Figure 4:**
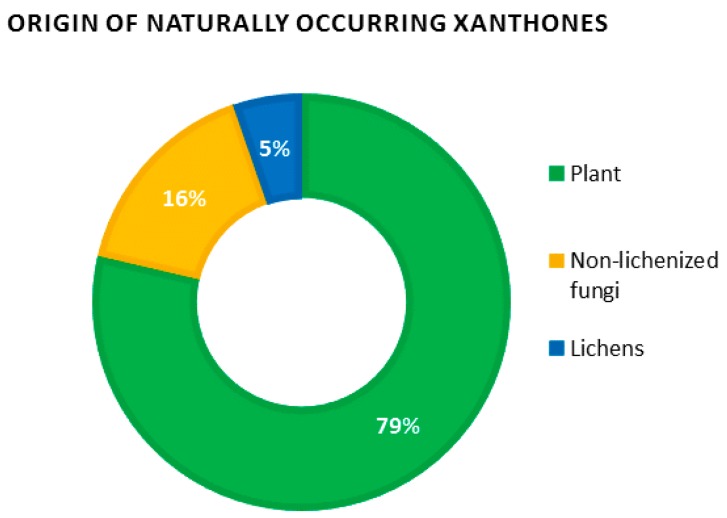
Pie diagram showing the distribution of source organisms for 1940 naturally occurring xanthones (established by consultation of the Dictionary of Natural Products, 15 January 2016).

Recent data regarding the number of naturally occurring xanthones are scarce, with the last numbered record of 278 xanthones listed by Vieira and Kijjoa more than 10 years ago [3]. By January 2016, the Dictionary of Natural Products revealed a dramatic increase in the number of natural xanthones with *ca*. 2000 occurrences of xanthones *sensu lato* (*i.e.*, including their reduced derivatives di-, tetra- and hexahydroxanthones). These considerable efforts might be explained by the discovery of promising leads such as the anti-angiogenic molecule Vadimezan (AS 404) which is currently undergoing phase III clinical trials as a tumor vascular-disrupting agent [35,36], as well as the pleiotropic pharmacological activities of mangosteen xanthones which have garnered considerable interest from the scientific community [37,38]. Obviously, plants remain the prevalent source of xanthones, concentrating almost 80% of natural xanthones. Non-lichenized fungi represent a further 15% while lichens account for the remaining 5% (Figure 4).

Structural diversity of lichen xanthones mostly stems from variations in the orientation and degree of chlorination of the norlichexanthone or ravenelin core, as well as from the position and extent of methylation of the phenolic groups. Elix and Crook tremendously advanced the understanding of chlorination and methylation processes affecting the xanthone core. Through the example of 40 different species of lichens, theoretical biosynthetic schemes mirroring the sequence of biogenetic events leading to the joint occurrence of xanthones among lichens were constructed [39]. This results in chemosyndromes in which the biosynthetic intermediates can be expected alongside the final xanthone. As an example, isoarthothelin (=2,5,7-trichloronorlichexanthone) might be biosynthesized from 2,4-dichloronorlichexanthone and 2,5-dichloronorlichexanthone. Therefore, the joint occurrence of these species is referred to as the isoarthothelin chemosyndrome [40]. 

Further structural modifications might be taken into account to extend the chemodiversity of lichen xanthones. Important contributors to the chemical diversity of xanthones are prenyl groups which are most often incorporated as dimethylallyl moieties [41]. Generally speaking, prenylated secondary metabolites are of paramount interest since their bioactivities are often distinct from those of their non-prenylated precursors [42]. Even though prenylxanthones have been known to come from fungi for a long time [43], no reports of such xanthones were made by the time Huneck and Yoshimura published their compendium of lichen substances [5] since the first lichen prenylxanthones were reported by Rezanka *et al.* in 2002 [44]. Underlying biosynthetic pathways were recently outlined within the prenylxanthone-producing fungus *Aspergillus nidulans*, through the identification of prenyltransferase genes belonging to the fungal indole prenyltransferases, so far known for their involvement in the prenylation of amino acids [45]. Glycosylations represent another kind of functionalization recently evidenced from lichen xanthones through the examples of umbilicaxanthosides A and B [44] and hirtusneanoside [46]. 

The diversity of lichen xanthones is also extended through some dimeric xanthones. Although the identification of key dimerization processes still warrants further investigation, xanthone dimers are most likely obtained after the biaryl linkage of monomers. This hypothesis is preferred to that of a tandem biosynthetic pathway which would involve a side-by-side cyclization of a double-length polyketide, especially since the discovery of the long-sought-after monomeric units of secalonic acids (the so-called blennolides) within the fungus *Blennoria* sp. [47]. Enzymatically mediated or not, it is admitted that the dimerization would involve a xanthonyl radical that subsequently couples to electron donors. Resonance contributors of this delocalized aryl radical might then account for the reactivity of *ortho* and *para* C positions [24].

Reductive dearomatizations are sometimes observed on xanthones to yield dihydro-, tetrahydro- or hexahydroxanthones. To date, in lichens, such reduced species were only observed from dimeric xanthones: secalonic acids, hirtusneanoside and eumitrin A1 (bis tetrahydroxanthones) and eumitrin A2, B and T (unsymmetrical tetrahydro- and hexahydroxanthones). Overall, a vast majority of xanthones reported from lichens have a monomeric and fully aromatized structure.

## 3. Contribution of the Symbiotic Partners

Even though xanthones from free-living fungi are well known, a plant-fungus collaboration has been suggested for several lichen xanthones. As an example, the typical lichen xanthone 2,7-dichlorolichexanthone could be isolated from the lichen *Lecanora dispersa* [48]. However, when the fungus was cultivated in the absence of the alga, the xanthone production was diverted to other secondary metabolites being produced instead (e.g., depsidones such as pannarin and related compounds). A further consistent example is that of *Lecanora rupicola*. This lichen is known to produce metabolites of various polyketide classes: lecanoric acid (depsides), hematommic and orsellinic acids (monocyclic phenols), eugenitol and sordidone (chromones) and arthothelin (xanthone). The whole chemosyndrome could be produced from axenically grown mycobionts, with the notable exception of arthothelin [49], suggesting a likely metabolic cooperation between the symbiotic partners. Likewise, dimeric xanthones eumitrins are produced by the lichen *Physconia distorta* but not by its isolated mycobiont [50]. Adversely, axenic cultures of the mycobiont of *Pyrenula japonica* and *P. pseudobufonia* revealed the biosynthesis of xanthones that cannot be evidenced from the lichen as a whole (see further), suggesting their significance in the pre-lichenized condition [51,52].

A total of 72 xanthones containing one to four chlorine atoms are currently known from lichens. Even though recent studies have shed light on chlorinated xanthone biosynthesis, no study has focused on their degradation. Dechlorination of organochlorines can be achieved by some bacterial strains, referred to as organohalide respirers. Such bacteria comprise the famous *Dehalococcoides mccartyi* that has gathered attention because of its ability to dechlorinate a large array of anthropogenic pollutants [53,54,55]. Later on, related bacteria were also reported from an unpolluted environment, suggesting their involvement in homeostatic chlorine cycling [56,57,58]. Therefore, a contribution from the rich bacterial communities sheltered by lichens can be envisaged. Notably, some such organohalide respirers belong to the Firmicutes phylum [59], which is represented among the bacterial diversity hosted by lichens [60,61,62]. However, comparative studies of bacterial populations harvested by chlorinated xanthones producing lichens and lichens lacking such metabolites are mandatory for validating this assumption. Given the close structural homologies between chlorinated xanthones and polychlorinated biphenyls (PCB) and dibenzo-*p*-dioxins (dioxins), such bacterial strains might be of paramount interest for bioremediation purposes [59]. Acting as alternative electron acceptors, chlorinated xanthones might serve as biostimulants to speed up the remediation at contaminated sites [59,63].

## 4. Analytical Chemistry: From Detection to Structure Elucidation

**Figure 5:**
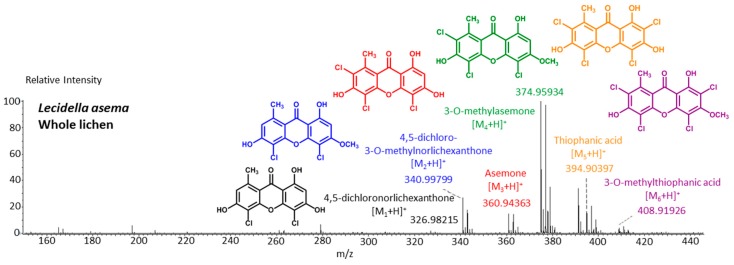
Positive-ion mode DART-MS spectrum of a solid piece of *Lecidella asema*. Experimental conditions as described in [80].

Although widespread among lichens, the long neglect of xanthones compared to other structural classes most likely arises from initial confusion in structural assignments between isomers and the subsequent difficulties in distinguishing the large number of co-occurring isomers [64]. However, the reliability of structural assignments tremendously increased over the last decades as refined dereplicative processes were developed. By 1993, a normalized reverse phase HPLC technique defined retention indices for 393 lichen products—including 55 xanthones—revealing that numerous xanthone isomers had different retention times [65]. Likewise, standardized TLC procedures were defined [66,67,68], with specific guidelines dedicated to xanthones [69]. A further enhancement of the normalized HPLC procedure was its subsequent hyphenation with a photodiode array detector to screen for xanthones based on their specific UV/Vis spectra [70]. As fluorescent substances, the color of lichen metabolites in long-wave UV light has been used for a long time as a hint to determine their structural class, especially for TLC. While the color of xanthones at such wavelengths is expected to range from bright yellow to orange, anthraquinones vary from bright red to vermillion and pulvinic acid derivatives appear yellowish [71]. At last, depsides and depsidones generally fluoresce blue to white or shades of grey, although atranorin gives a yellow hue [72]. Such features paved the way for fluorescence microscopy studies undertaken on semi-thin sections of lichens to explore the location of lichen metabolites across sections of a range of macrolichens [73]. However, an obvious limitation of this approach is that it only depends on the structural group scaffold and therefore it does not discriminate between individual compounds. Structural assignments have now been confirmed by the unambiguous synthesis of most lichen xanthones [74,75,76]. A further strategy to improve the information granted by the aforementioned separative techniques is the subsequent detection by mass spectrometry. If HPLC-MS of lichen extracts is now widely used for lichen dereplication, with the possible detection of xanthones, no TLC-MS reports have yet been made to the best of the authors’ knowledge [77]. To date, no standardized liquid chromatography-UV-mass spectrometry methodology has been published on lichen metabolites. However, a few non-specific lichen xanthones (lichexanthone, norlichexanthone, secalonic acid D) can be found in the fungal database established by Nielsen and Smedsgaard [78]. As far as mass spectrometry is concerned, various lichen xanthones were shown to be ionizable in negative-ion mode LDI-MS (Laser Desorption and Ionization), directly providing a complete chemical fingerprint from the complete acetone extract of the chloroxanthone-containing *Lecidella asema*, without the need for matrix assistance [79]. Lately, the ambient ionization technique DART-MS revealed its potent ability in examining lichen secondary metabolites *in situ* [80]. As shown below, the analysis of a whole piece of *Lecidella asema* shows an exhaustive chemical profile (Figure 5).

However, regarding the specific example of lichen xanthones, the lack of mutually supportive data such as chromatographic retention times and/or UV/Vis spectra precludes the differentiation of isomers and is a severe limitation of such methods. Possible ways to overcome such limitations while keeping an *in situ* approach might be the use of a droplet microjunction surface sampling probe and subsequent coupling with HPLC-DAD-MS(MS) to perform separation and therefore to collect retention time and UV/Vis data as obtained with more traditional dereplication protocols [81]. Even though such enhancements might shorten the time lapse for drug discovery through pinpointing and streamlining the isolation of original compounds, NMR analysis remains mandatory for elucidating structure, especially for highly isomeric structures such as xanthones. For a long time, the poor sensitivity of NMR precluded the structure elucidation of numerous minor to trace compounds. However, since 2000, significant improvements in reducing the operational sample amount and concentration addressed this shortcoming, especially through the use of low-volume tube probes and capillary probes coupled with cryogenically cooled radiofrequency coils [82]. Such innovations pave the way for the full characterization by one-dimensional (1D) and two-dimensional (2D) NMR of natural products in vanishingly small amounts (down to one nanomole) in reasonable time frames [82,83]. This increased sensitivity is of special significance in the field of lichenology since (i) the collection of bulk quantities of material is often hard to achieve and (ii) the high degree of accumulation of the major metabolites in lichens remains a major pitfall when studying their phytochemistry [84].

Together with analytical achievements, informatics tools were also developed to alleviate the dereplication holdup. A computer software named Wintabolites^®^ was dedicated to the identification of lichen metabolites from an original database of 550 compounds, including HPLC retention indices, TLC-Rf values, UV/visible colors of TLC spots and thalline reactions to guide their identification [85]. A most valuable upgrade of this informatics approach is LIAS metabolites (A Global Information for Lichenized and non-lichenized Ascomycetes) [86], a database compiled by Pr. J.A. Elix with additions by Pr. K. Kalb. Extended to 881 secondary metabolites as of January 2016, this database now includes mass spectrometry data besides HPLC and TLC standardized chromatographic values. Useful information regarding the structural class of the metabolites and biosynthetically related compounds is also given [87].

## 5. Structures and Bioactivities of Lichen Xanthones

It is noteworthy that lichen xanthones described up to 1996 most often belonged to the two basic cores described earlier with canonical substitution patterns. Today, 62 molecules displaying the lichexanthone scaffold and 19 molecules following the thiomelin substitution pattern (Figure 2) have been described. However, the logical biosynthetic sequences delineated by Elix and Crook [39] enable the prediction of putative intermediates that have not yet been reported from lichens. These putative intermediates might represent eight additional lichexanthone derivatives and three other thiomelin-type xanthones. Interestingly, a number of xanthone species described as putative in 1996 [5] have been purified since then. Such species include 7-chlorolichexanthone (*Lecanora schofieldii* [88]), 2,4-dichloro-3-*O*-methylnorlichexanthone (*Pertusaria aceroae* and *P. calderae* [89]), 2,5-dichloro-3-*O*-methylnorlichexanthone (obtained from *Calopadia fusca* [90] among others), 3,6-di-*O*-mehtylthiophanic acid from *Calopadia subcoerulescens* [90] and *Phyllopsora chodatinica* [91]), 4,5,7-trichlorolichexanthone (*Sporopodium leprosum* [92]) and 2,4,7-trichloro-3-*O*-methylnorlichexanthone (*Calopadia perpallida* [90]). Chlorinations and methylations from these cores can directly account for the structures of most lichen xanthones described by Huneck and Yoshimura. This diversity of lichen xanthones has to be extended to the vast array of secalonic acid and eumitrin derivatives identified by their chromatographic data but for which their minute amounts have so far hampered their NMR structure elucidation. Such tetrahydroxanthone dimers might represent privileged metabolites given their structural homology with the promising anti-tumor compound phomoxanthone A [93]. One might also take into account the putative monomeric units of secalonic acids and eumitrins, none of them being identified from lichen sources so far.

**Figure 6:**
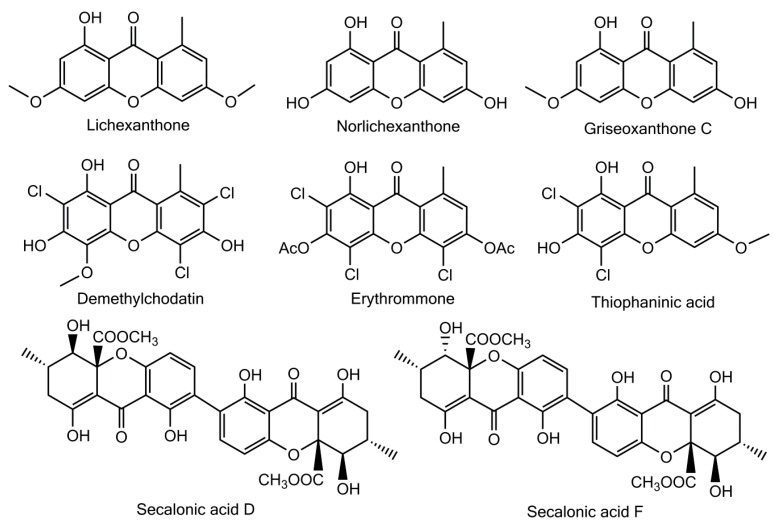
Chemical structures of xanthones with trivial names discussed in this manuscript.

Besides these fully rationalized structures, a limited number of metabolites exhibit structural variations that are more or less difficult to explain. As an example, demethylchodatin displays a methoxy group in position 4 that might be introduced by a xanthone oxidase on this trichlorinated norlichexanthone derivative. Another unusual modification of the lichexanthone series is the occurrence of two acetyl groups in erythrommone. The chemical structures of xanthones with trivial names discussed in this manuscript are enlisted in Figure 6.

However, 22 lichen xanthones display unusual structural features that make their biosynthetic intermediates tricky to unravel (including a series of 16 related species described by Rezanka and colleagues [44,94], see further). Three such species were already mentioned in the compendium established by Huneck and Yoshimura [5]: the absence of a methyl substituent in 1,8-dihydroxy-3,6-dimethoxyxanthone is biogenetically difficult to explain, as are the two methyl substituents of 1,7-dihydroxy-2,4-dichloro-6,8-dimethylxanthone and of 1-hydroxy-2,4-dichloro-6,8-dimethylxanthone, obtained from *Rinodina thiomela*.

As of 2016, a total of 103 xanthones were isolated from lichens, implying the presence of 11 further species as biosynthetic intermediates. This chemodiversity will soon be enriched with an extensive array of unidentified tetrahydrobisxanthones and their associated monomeric units.

Very few lichen xanthones have been investigated to date for their bioactivities. Lichexanthone exerts a weak activity against *Mycobacterium tuberculosis* [95] and *M. aurum* [96]. Despite this mild activity, a dihydropyrane xanthone derivative of lichexanthone revealed an antimycobacterial activity comparable to that of drugs commonly used to treat tuberculosis [97]. Lichexanthone and griseoxanthone C exhibit strong antibiotic effects towards *Bacillus subtilis* with respective IC_50_ values of 2.25 and 1.29 μM, but only the former inhibited the growth of methicillin-resistant *Staphylococcus aureus* (IC_50_: 21 μM) [22]. No anti-parasitic activity of lichexanthone could be observed towards *Plasmodium* *falciparum* and *Trypanosoma brucei* [15]. Lichexanthone also displays sperm mobility–enhancing properties [98] and induces the production of NO by murine macrophages which might reveal their activation [99]. Lichexanthone revealed no cytotoxic activity against murine melanoma B16F10, human melanoma UACC-62 and fibroblast cells NIH/3T3 [100]. At last, lichexanthone was found to be effective against the dengue vector *Aedes aegypti* [98].

Norlichexanthone was shown to display promising cytotoxic activities [101]. Indeed, norlichexanthone caused 100% inhibition of p56^lck^ tyrosine kinase at 200 μg/mL [102] and inhibited the activity of the protein kinases aurora-B, PIM1 and VEGF-R2 with mean IC_50_ values ranging from 0.3 to 12 μM [103]. Norlichexanthone also promotes the expression of the insulin-sensitizing, anti-diabetic and anti-atherogenic protein adiponectin within cultured ST-13 adipocytes [104]. Dayan and Romagni also reported on the fungicidal effect of thiophanic (Figure 5) and thiophaninic acids [105]. Alongside many other lichen compounds, Huneck and Schreiber reported on the allelopathic effect of thiophanic acid towards a wide array of higher plants [106].

As most structural classes of lichen metabolites, xanthones display strong UV-absorbing properties, predominantly in the wavelength range of UVA [107]. Indeed, lichexanthone synthesis could be triggered in juvenile mycelia of *Haematomma fluorescens* as a response to a 365 nm UV light exposure, emphasizing its protective role as a light filter [108]. Deposition of xanthones in the cortical layers further suggests their involvement in the protection of the UV-sensitive algal layer [109,110]. Such properties might be of utmost interest when urgently needing new materials to outperform the currently used UV-photoprotective products [111]. A Time-Dependent Density Functional Theory (TD-DFT) modelization recently delineated the electronic transitions accounting for the UV/Vis spectra of secalonic acids. This revealed that absorption wavelengths and molecular extinction coefficients obtained from these bisxanthones were comparable to that of UVA-referent sunscreens, which might stem from the structural homologies shared by the commercially available avobenzone and the phenyl-β-diketo moiety of xanthones [112].

## 6. New Lichen Xanthones Described since 1996: Discussion, Physico-Chemical Properties (Including NMR Data) and Bioactivities

The past 20 years witnessed the isolation of 23 further lichen xanthones. It is noteworthy that those new xanthones displayed original substitution patterns and revealed original moieties compared to the previous state of the art.

Cultures of the spore-derived mycobionts of *Pyrenula japonica* and *P. pseudobufonia* afforded a series of five new ravenelin-type lichen xanthones, the first reports of such xanthones from aposymbiotically grown lichen mycobionts. A first report elucidated the structures of 1,5,8-trihydroxy-3-methylxanthone, 1,8-dihydroxy-5-methoxy-3-methylxanthone and 1,7-dihydroxy-3-methylxanthone [51] while a second one established the occurrence of 1,8-dihydroxy-3-hydroxymethyl-5-methoxyxanthone and 1,4,8-trihydroxy-5-methoxy-3-methylxanthone, typically associated with the thiomelin pathway [52]. Notably, 1,7-dihydroxy-3-methylxanthone stands among the rare xanthones also described from plants (*Cassia occidentalis* [113]) and also from an Ascomycete fungus [114]. Surprisingly, all molecules described from this series lack the 4-hydroxy group with four of them displaying an unusual oxygenated substituent at C5 instead. In one case, position 2 is oxygenated, which is a unique structural feature among the lichen xanthones described so far. One will note that 1,7-dihydroxy-3-methylxanthone also lacks the oxygenated substituent on C8 which is replaced by a unique hydroxyl substituent on C7, an unprecedented structural peculiarity for a lichen xanthone. Finally, the oxidation of the methyl group into a hydroxymethyl for one of these molecules is also new to lichen xanthones. Of special interest is that these compounds could not be identified from the whole lichen, whereas they are constantly isolated from mycobionts harvested in highly unrelated sampling sites (one in Japan and one in the USA), which might point to their biological significance in the prelichenized condition. Notably, 1,5,8-trihydroxy-3-methylxanthone and 1,2,8-trihydroxy-5-methoxy-3-methylxanthone displayed stronger antioxidant activities than α-tocopherol via the DPPH radical test [52]. Besides, 1,7-dihydroxy-3-methylxanthone also exerts a moderate Mono Amine Oxidase-inhibiting activity [114].

Two highly unusual glycosylated prenylxanthones were subsequently identified from the Ural lichen *Umbilicaria proboscidea* [44]. Umbilicaxanthoside A is a C2-monoprenyl xanthone having a β-d-glucopyranose unit anchored at position O-7, whereas umbilicaxanthoside B stands for a C2,C8-diprenylxanthone with a disaccharide β-d-glucopyranose-(1→4)-β-d-glucopyranose moiety at *O*-7. These compounds were the first prenylated xanthones to be isolated from a lichen source and glycosides are not frequently encountered in lichens [84]. Besides, both molecules reveal a highly unusual oxygenation pattern, rendering their biosynthetic pathway tricky to delineate. A follow-up study carried out on the same lichen identified 14 acylated xanthone *O*-glycosides corresponding to linolenoyl, lineoyl, palmitoleoyl, oleoyl, palmitoyl, eicosenoyl and stearoyl esters of both umbilicaxanthosides A and B [94]. Fragmentation patterns obtained by LC-APCI-MS were highly informative and could establish the nature of the esterified fatty acid. None of those compounds were investigated for their biological properties.

**Figure 7:**
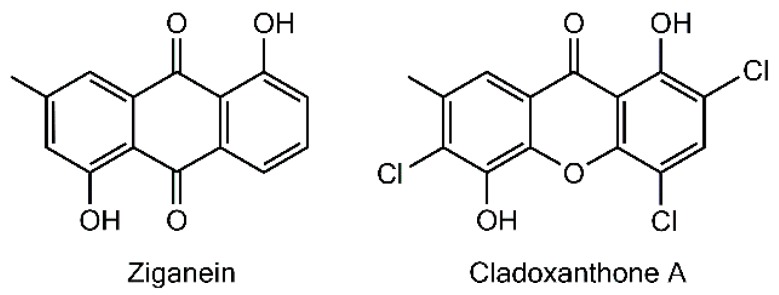
Compared chemical structures of ziganein and cladoxanthone A.

The last monomeric xanthone isolated so far from a lichen source is cladoxanthone A (=1,5-dihydroxy-2,4,6-trichloro-7-methylxanthone), obtained from *Cladonia incrassata* [115]. The occurrence of a methyl substituent on C-7 is an unprecedented structural feature among lichen xanthones, thus questioning the underlying biogenetic schemes. A possible hypothesis to account for the structure of this xanthone is to consider ziganein as a possible anthraquinone precursor [116]. It is noteworthy that this metabolite presents a substitution pattern identical to that of cladoxanthone A and it was isolated from the endophytic fungus *Sporormiella minimoides* [116] (Figure 7). Cladoxanthone A revealed an antibacterial effect towards *Staphylococcus aureus* even though the paucity of the compound precluded the determination of a minimal inhibitory concentration [115].

Regarding dimeric xanthones, known metabolites of the secalonic acid series were first isolated from lichen sources in 2009, *i.e.*, secalonic acids B, D and F were purified from *Diploicia canescens* [117]. The joint occurrence of these isomers in this lichen is not surprising because of their biosynthetic relationship. Indeed, the association of the two monomeric tetrahydroxanthone blennolides A and B, respectively, yields secalonic acids B and D [47]. Secalonic acid F then represents a hybrid dimer of blennolides A and B. It is noteworthy that each of these compounds displays an identical configuration at C-10a, suggesting that the cyclization is catalyzed by a specific enzyme giving rise to a unique type of product [117]. Secalonic acid is a major environmental mycotoxin essentially known for its production by the microbial *Penicillium oxalicum*, a notorious foodstuff contaminant. Such a presence represents an alarming health issue given the acute toxicity and the teratogenic effects of secalonic acid D [118,119], warranting extended investigations of its bioactivities which highlighted its pleiotropic pharmacological activities. Secalonic acids D and F revealed antimicrobial activities against *Bacillus megaterium* while secalonic acid A displayed activity against *Bacillus subtilis* and *Piricularia oryzae* [120]. Likewise, secalonic acid B is an effective antimicrobial (*Bacillus megaterium* and *Escherichia coli*) as well as antifungal (*Microbotryum violaceum*) and antialgal agent (*Chlorella fusca*) [47]. Several reports suggested the cytotoxic activity of secalonic acid derivatives [117]. Bioassay-guided fractionation of the extracts of the marine lichen–derived *Gliocladium* sp. T 31 streamlined the isolation of secalonic acid D as the cytotoxic metabolite against four cell lines in a highly variable range of 0.03–15 μM, suggesting selective pharmacodynamic properties [121]. Further studies revealed a submicromolar IC_50_ of secalonic acid D on the carcinoma KB cells and an inhibition of human topoisomerase 1 with a promising IC_50_ of 0.16 μg/mL [122]. More recently, secalonic acid D was shown to down-regulate the expression of efflux pump ABCG2 which is known to confer the Multidrug Resistance phenotype [123]. As a last contribution to its cytotoxic activity, secalonic acid D exhibits an anti-angiogenic activity via the Akt/mTor/P70S6K pathway [124]. Another feature of the pleiotropic activities of secalonic acid D depends on the inhibition of protein kinase C and several other Ca^++^-dependent enzymes through competitive inhibition [125]. Subsequent disruptions of cell signaling might account for the teratogenicity of these compounds. Indeed, it was demonstrated on a murine model that secalonic acid D decreases the levels of Epidermal Growth Factor (EGF) as well as its associated signal transduction [126]. As a consequence, inhibition and alteration of transcription factors in the developing murine plate following an exposition to the toxin at normal human dietary levels were observed, resulting in the cleft palate condition [127,128].

Hirtusneanoside is a new xanthone dimer isolated from *Usnea hirta* corresponding to a rhamnoside of an unsymmetrical dimeric tetrahydroxanthone [46]. If the oxygenation pattern can be fully rationalized according to the biosynthetic pathway discussed earlier, this structure contains several additional methyl groups of unknown origin. Hirtusneanoside was proven to be active against *Staphylococcus aureus* and *Bacillus subtilis* [46]. The enzymatic hydrolysis of hirtusneanoside yielded α-l-rhamnose and the aglycone named hirtusneanin, the physico-chemical data of which were also reported by Rezanka and Sigler [46].

Available physico-chemical properties regarding the newly described lichen compounds are listed below.

**Cladoxanthone A**

C_14_H_7_O_4_Cl_3_ (343.94099)

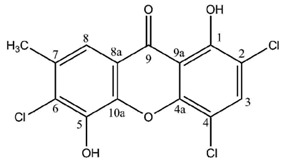


Yellow powder

UV λ_max_ (MeOH)(log ε): 381 (3.24), 323 (3.50), 259 (4.11) nm

IR ν_max_ (CHCl_3_): 3353, 3056, 1642, 1597, 1450, 800 cm^−1^

Sources: *Cladonia incrassata* [115].

**Table:** 

Cladoxanthone A (CDCl_3_)		
Position	δ_C_	δ_H_ (*J* in Hz)
1	156.2	-
–OH 1	-	13.16 (s) (1H)
2	115.2	-
3	136.6	7.80 (s) (1H)
4	111.2	-
4a	149.5	-
5	141.2	-
–OH 5	-	6.09 (s) (1H)
6	127.9	-
7	133.7	-
CH_3_–7	20.2	2.51 (s)
8	117.0	7.73 (s)
8a	118.4	-
9a	109.7	-
10a	143.0	-

**1,8-Dihydroxy-3-hydroxymethyl-5-methoxyxanthone**

C_15_H_12_O_6_ (288.06339)

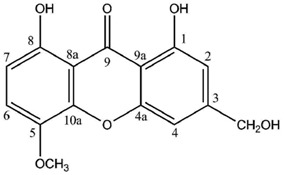


Yellow needles, mp 221–222 °C

UV λ_max_ (MeOH)(log ε): 387.5 (3.27), 341 (3.84), 271 (4.16), 263 (4.24), 255 (4.29), 236 (4.17)

IR ν_max_ (KBr): 3533, 1659, 1634, 1609, 1589, 1493 cm^−1^

Sources: Mycobiont of *Pyrenula japonica* [52].

**Table:** 

1,8-Dihydroxy-3-hydroxymethyl-5-methoxyxanthone (DMSO-*d*_6_)		
Position	δ_C_	δ_H_ (*J* in Hz)
1	160.0	-
–OH 1	-	11.10 (br s) ^a^
2	107.7	6.98 (br s) (1H)
3	154.8	-
3–CH_2_OH	62.2	4.57 (br s) (2H)
3–CH_2_OH	-	5.58 (br s) (1H)
4	104.2	6.75 (br s) (1H)
4a	155.6	-
5	139.7	-
5–OCH_3_	56.7	3.86 (s) (3H)
6	121.1	7.45 (d, 9.0) (1H)
7	108.9	6.71 (d, 9.0) (1H)
8	152.7	-
8–OH	-	11.60 (br s) ^a^
8a	107.8	-
9	184.9	-
9a	106.1	-
10a	144.8	-

^a^ These signals might be switched.

**1,2,8-Trihydroxy-5-methoxy-3-methylxanthone**

C_15_H_12_O_6_ (288.06339)

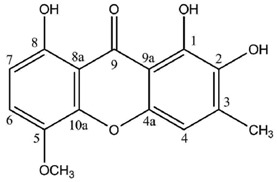


Yellow needles, mp 214–215 °C

UV λ_max_ (MeOH) (log ε): 421 (3.42), 352 (3.58), 280 (4.27), 263 (4.18), 243 (4.17), 206 (4.23)

IR ν_max_ (KBr): 3487, 1663, 1636, 1607, 1576, 1489 cm^−1^

Sources: Mycobiont of *Pyrenula japonica* [52].

**Table:** 

1,2,8-Trihydroxy-5-methoxy-3-methylxanthone (DMSO-*d*_6_)		
Position	δ_C_	δ_H_ (*J* in Hz)
1	145.8	-
2	138.4	-
–OH 2	-	
3	137.0	-
3–CH_3_	17.1	2.30 (s) (3H)
4	107.6	6.95 (br s) (1H)
4a	147.7	-
5	139.7	-
5–OCH_3_	56.7	3.88 (s) (3H)
6	120.5	7.45 (d, 9.0) (1H)
7	108.2	6.70 (d, 9.0) (1H)
8	152.6	-
8a	107.6	-
9	185.1	-
9a	105.9	-
10a	145.0	-

**1,7-Dihydroxy-3-methylxanthone**

C_14_H_10_O_4_ (242.05791)

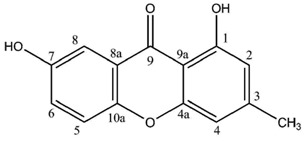


Yellow needles, mp 259–260 °C

UV λ_max_ (MeOH) (log ε): 384 (3.84), 290 (3.98), 261 (4.58), 235 (4.45)

IR ν_max_ (KBr): 3285, 1653, 1607, 1585, 1483 cm^−1^

Sources: Mycobiont of *Pyrenula japonica* [52].

**Table:** 

1,7-Dihydroxy-3-methylxanthone (CDCl_3_-CD_3_OD)		
Position	δ_C_	δ_H_ (*J* in Hz)
1	161.7	-
2	111.1	6.57 (br s) (1H)
3	149.5	-
3–CH_3_	22.6	2.42 (s) (3H)
4	108.1	6.75 (br s) (1H)
4a	157.1	-
5	119.6	7.37 (d, 9.0) (1H)
6	125.5	7.29 (dd, 9.0, 2.5) (1H)
7	154.5	6.70 (d, 9.0) (1H)
8	109.0	7.52 (d, 2.5) (1H)
8a	121.6	-
9	182.5	-
9a	107.1	-
10a	150.8	-

**1,5,8-Trihydroxy-3-methylxanthone**

C_14_H_10_O_4_ (258.05282)

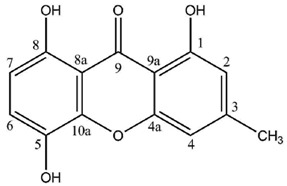


Yellow needles, mp 277–278 °C

UV λ_max_ (MeOH) (log ε): 403 (3.54), 341 (4.01), 272 (4.58), 263 (4.40), 255 (4.47), 236 (4.32)

IR ν_max_ (KBr): 3461, 1661, 1633, 1606, 1591, 1497 cm^−1^

Sources: Mycobiont of *Pyrenula japonica* and *Pyrenula pseudobufonia* [52].

**Table:** 

1,5,8-Trihydroxy-3-methylxanthone (CDCl_3_)		
Position	δ_C_	δ_H_ (*J* in Hz)
1	161.4	-
1–OH	-	11.85 (s) (1H) ^a^
2	111.9	6.62 (m) (1H)
3	150.6	-
3–CH_3_	22.7	2.45 (s) (3H)
4	108.5	6.89 (m) (1H)
4a	156.9	-
5	137.8	-
6	124.6	7.24 (d, 9.0) (1H)
7	110.0	6.64 (d, 9.0) (1H)
8	153.6	-
8–OH	-	11.27 (br s) (1H) ^a^
8a	108.6	-
9	186.5	-
9a	106.4	-
10a	144.7	-

^a^ Signals appearing in DMSO-*d*_6_ only.

**1,8-Dihydroxy-5-methoxy-3-methylxanthone**

C_15_H_12_O_5_ (272.06847)

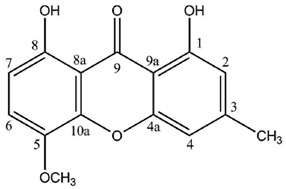


Yellow needles, mp 214–215 °C

UV λ_max_ (MeOH) (log ε): 393 (3.54), 340 (4.00), 272 (4.30), 254 (4.49), 235 (4.36)

IR ν_max_ (KBr): 3445, 1661, 1631, 1609, 1585, 1489 cm^−1^

Sources: Mycobiont of *Pyrenula japonica* and *Pyrenula pseudobufonia* [52].

**Table:** 

1,8-Dihydroxy-5-methoxy-3-methylxanthone (CDCl_3_)		
Position	δ_C_	δ_H_ (*J* in Hz)
1	161.0	-
1–OH	-	11.73 (s) (1H) ^a^
2	111.7	6.62 (br s) (1H)
3	149.9	-
3–CH_3_	22.6	2.42 (s) (3H)
4	108.0	6.84 (br s) (1H)
4a	156.0	-
5	140.0	-
5–OCH_3_	57.4	3.94 (s) (3H)
6	120.8	7.23 (d, 9.0) (1H)
7	109.1	6.70 (d, 9.0) (1H)
8	154.2	-
8–OH	-	11.33 (br s) (1H) ^a^
8a	108.3	-
9	185.6	-
9a	105.9	-
10a	145.6	-

^a^ Signals appearing in DMSO-*d*_6_ only.

**Hirtusneanoside**

C_40_H_46_O_17_ (798.27350)

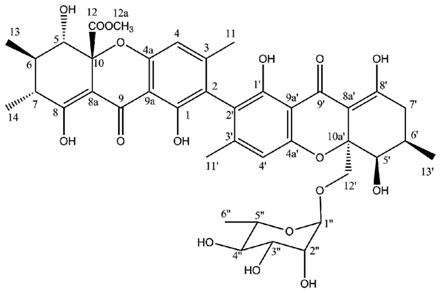


Faint yellow crystals, mp 231–232 °C

${\left[\alpha \right]}_{D}^{20}$ = −251 (*c* = 0.02 in MeOH)

UV λ_max_ (MeOH)(log ε): 340 (3.24), 275 (4.01), 230 (4.52) nm

IR ν_max_ (CHCl_3_): 3290, 1735, 1620, 1590, 870 cm^−1^

Sources: *Usnea hirta* [46].

**Table:** 

Hirtusneanoside (DMSO-*d*_6_)		
Position	δ_C_	δ_H_ (*J* in Hz)
1	159.2	-
–OH 1	-	11.5 (br s) (1H)
2	116.7	-
3	149.6	-
4	109.2	6.66 (s) (1H)
4a	156.8	
5	68.8	4.02 (d, 9.5) (1H)
6	31.3	2.05 (ddq, 10.3, 9.5, 6.7) (1H)
7	36.7	2.36 (dq, 10.3, 6.4) (1H)
8	177.8	-
–OH 8	-	13.7 (br s) (1H)
8a	101.3	-
9	186.8	-
9a	105.7	-
10a	85.1	-
11	20.7	1.94 (s) (3H)
12	171.3	-
12a	54.3	3.73 (s) (3H)
13	15.6	1.09 (d, 6.7) (3H)
14	17.3	1.01 (d, 6.4) (3H)
1′	159.6	-
1′–OH	-	11.5 (br s) (1H)
2′	118.1	-
3′	150.2	-
4′	109.3	6.69 (s) (1H)
4a′	156.7	-
5′	68.9	4.07 (d, 1.3) (1H)
6′	28.5	2.28 (dddq, 11.3, 6.7, 6.5, 1.3) (1H)
7′	33.6	2.48 (dd, 19.2, 11.3) (1H)
		2.35 (dd, 19.2, 6.5) (1H)
8′	177.6	-
8′–OH	-	13.7 (br s) (1H)
8a′	101.8	-
9′	186.6	-
9a′	106.3	-
10a′	84.4	-
11′	20.6	1.96 (s) (3H)
12′	64.5	3.89 (d, 13.0) (1H)
		3.51 (d, 13.0) (1H)
13′	17.7	1.04 (d, 6.7) (3H)
1″	101.1	5.01 (d, 1.5) (1H)
2″	72.2	3.89 (dd, 2.5, 1.5) (1H)
3″	70.9	3.71 (dd, 9.5, 2.5) (1H)
4″	74.5	4.32 (t, 9.5) (1H)
5″	69.7	4.13 (dq, 9.5, 6.5) (1H)
6″	18.6	1.31 (d, 6.5) (3H)

**Hirtusneanine**

C_34_H_36_O_13_ (652.21559)

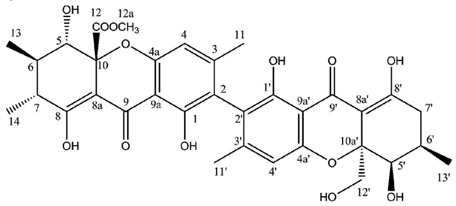


Pale yellow crystals, mp 251–253 °C

${\left[\alpha \right]}_{D}^{20}$ = −232 (*c* = 0.01 in MeOH)

UV λ_max_ (MeOH)(log ε): 338 (3.97), 275 (4.04), 231 (4.43) nm

IR ν_max_ (CHCl_3_): 3290, 1734, 1608, 1590, 870 cm^−1^

Sources: *Usnea hirta* [46].

**Table:** 

	Hirtusneanine (DMSO-*d*_6_)	
Position	δ_C_	δ_H_ (*J* in Hz)
1	159.2	-
–OH 1	-	11.5 (br s) (1H)
2	116.7	-
3	149.6	-
4	109.2	6.66 (s) (1H)
4a	156.8	
5	68.8	4.02 (d, 9.5) (1H)
6	31.3	2.05 (ddq, 10.3, 9.5, 6.7) (1H)
7	36.7	2.36 (dq, 10.3, 6.4) (1H)
8	177.8	-
–OH 8	-	13.7 (br s) (1H)
8a	101.3	-
9	186.8	-
9a	105.7	-
10a	85.1	-
11	20.7	1.94 (s) (3H)
12	171.3	-
12a	54.3	3.73 (s) (3H)
13	15.6	1.09 (d, 6.7) (3H)
14	17.3	1.01 (d, 6.4) (3H)
1′	159.6	-
1′–OH	-	11.5 (br s) (1H)
2′	118.1	-
3′	150.2	-
4′	109.3	6.69 (s) (1H)
4a′	156.7	-
5′	68.7	4.07 (d, 1.3) (1H)
6′	28.5	2.28 (dddq, 11.3, 6.7, 6.5, 1.3) (1H)
7′	33.6	2.48 (dd, 19.2, 11.3) (1H)
		2.35 (dd, 19.2, 6.5) (1H)
8′	177.6	-
8′–OH	-	13.7 (br s) (1H)
8a′	102.1	-
9′	186.6	-
9a′	106.3	-
10a′	84.1	-
11′	20.6	1.96 (s) (3H)
12′	68.7	4.14 (dd, 13.0, 7.0) (1H)
		3.75 (dd, 13.0, 4.7) (1H)
12′–OH		3.28 (m) (1H)
13′	17.7	1.04 (d, 6.7) (3H)

**Umbilicaxanthoside A**

C_25_H_28_O_11_ (504.16316)

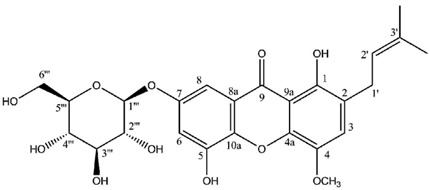


Yellow needles, mp 114 °C

${\left[\alpha \right]}_{D}^{23}$ = −35

UV λ_max_ (MeOH) (log ε): 345 (3.90), 310 (4.15), 270 (4.05), 245 (4.50).

IR ν_max_ (KBr): 3320, 2945, 2905, 1643 cm^−1^

Sources: *Umbilicaria proboscidea* [44].

**Table:** 

	Umbilicaxanthoside A (CDCl_3_)	
Position	δ_C_	δ_H_ (*J* in Hz)
1	150.9	-
1–OH	-	13.25 (s) (1H)
2	118.2	-
3	120.1	6.51 (s) (1H)
4	142.6	-
4–OCH_3_	56.4	3.75 (s) (3H)
4a	140.9	-
5	146.4	-
6	104.8	6.39 (d, 2.1) (1H)
7	153.0	-
8	108.1	6.73 (d, 2.1) (1H)
8a	128.9	-
9	179.8	-
9a	116.1	-
10a	136.5	-
1′	22.0	3.19 (d, 6.6) (2H)
2′	123.5	5.92 (td, 6.6, 1.3) (1H)
3′	131.3	-
4′	25.9	1.64 (s) (3H)
5′	17.9	1.57 (s) (3H)
1″	99.8	4.80 (d, 7.3) (1H)
2″	74.7	3.52 (dd, 8.9, 7.3) (1H)
3″	77.2	3.58 (t, 8.9) (1H)
4″	71.4	3.41 (t, 8.9) (1H)
5″	78.6	3.45 (m) (1H)
6″	62.6	3.93 (dd, 11.8, 2.3) (1H)
		3.72 (dd, 12.1, 5.2) (1H)

**Umbilicaxanthone A**

C_19_H_18_O_6_ (342.11034)

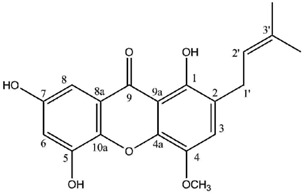


Sources: *Umbilicaria proboscidea* [44].

**Table:** 

	Umbilicaxanthone A (CDCl_3_)	
Position	δ_C_	δ_H_ (*J* in Hz)
1	150.9	-
1–OH	-	13.25 (s) (1H)
2	118.2	-
3	120.1	6.51 (s) (1H)
4	142.6	-
4–OCH_3_	56.4	3.75 (s) (3H)
4a	140.9	-
5	146.4	-
6	102.1	6.15 (d, 2.1) (1H)
7	156.9	-
8	106.3	^a^
8a	128.9	-
9	179.8	-
9a	116.1	-
10a	136.5	-
1′	22.0	3.19 (d, 6.6) (2H)
2′	123.5	5.92 (td, 6.6, 1.3) (1H)
3′	131.3	-
4′	25.9	1.64 (s) (3H)
5′	17.9	1.57 (s) (3H)

**Umbilicaxanthoside B**

C_36_H_46_O_16_ (734.27859)

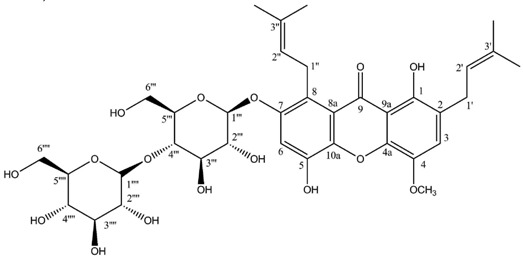


Pale yellow needles, mp 133 °C

UV λ_max_ (MeOH) (log ε): 355 (4.06), 319 (4.01), 271 (3.96), 244 (4.08)

IR ν_max_ (KBr): 3350, 2950, 2900, 1640 cm^−1^

Sources: *Umbilicaria proboscidea* [44].

**Table:** 

	Umbilicaxanthoside B (CDCl_3_)	
Position	δ_C_	δ_H_ (*J* in Hz)
1	151.0	-
1–OH	-	13.30 (s) (1H)
2	117.8	-
3	119.4	6.47 (s) (1H)
4	143.2	-
4–OCH_3_	56.3	3.75 (s) (3H)
4a	141.3	-
5	147.1	-
6	103.7	6.23 (s) (1H)
7	153.2	-
8	118.4	-
8a	129.6	-
9	183.1	-
9a	114.6	-
10a	135.7	-
1′	25.4	3.38 (d, 7.2) (2H)
2′	123.8	5.39 (td, 7.2, 1.2) (1H)
3′	130.9	-
4′	26.2	1.65 (s) (3H)
5′	18.4	1.79 (s) (3H)
1″	26.1	4.19 (d, 6.8) (2H)
2″	124.8	5.37 (td, 6.8, 1.5) (1H)
3″	132.0	-
4″	25.9	1.67 (s) (3H)
5″	18.0	1.84 (s) (3H)
1‴	99.8	5.05 (d, 8.1) (1H)
2‴	74.2	3.52 (dd, 8.9, 8.1) (1H)
3‴	76.9	3.58 (t, 8.9) (1H)
4‴	78.2	3.12 (dd, 9.3, 8.9) (1H)
5‴	78.7	3.45 (m) (1H)
6‴	62.6	3.93 (dd, 12.1, 2.3) (1H)
		3.72 (dd, 12.1, 5.2) (1H)
1’’’’	104.1	5.15 (d, 8.0) (1H)
2’’’’	73.8	3.52 (dd, 9.0, 8.0) (1H)
3’’’’	76.5	3.58 (t, 9.0) (1H)
4’’’’	69.7	3.41 (t, 9.0) (1H)
5’’’’	76.9	3.45 (m) (1H)
6’’’’	62.6	3.93 (dd, 12.1, 2.2) (1H)
		3.72 (dd, 12.1, 5.2) (1H)

**Umbilicaxanthone B**

C_24_H_26_O_6_ (410.17294)

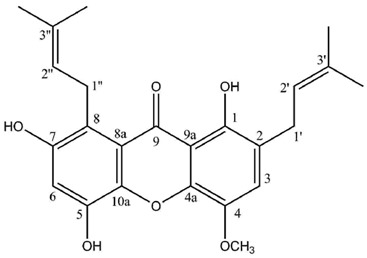


Sources: *Umbilicaria proboscidea* [44].

**Table:** 

	Umbilicaxanthone B (CDCl_3_)	
Position	δ_C_	δ_H_ (*J* in Hz)
1	151.0	-
1–OH	-	13.30 (s) (1H)
2	117.8	-
3	119.4	6.49 (s) (1H)
4	143.2	-
4–OCH_3_	56.3	3.75 (s) (3H)
4a	141.3	-
5	146.2	-
6	102.4	6.27 (s) (1H)
7	157.2	-
8	117.6	-
8a	129.6	-
9	183.1	-
9a	114.6	-
10a	135.7	-
1′	25.4	3.38 (d, 7.2) (2H)
2′	123.8	5.39 (td, 7.2, 1.2) (1H)
3′	130.9	-
4′	26.2	1.65 (s) (3H)
5′	18.4	1.79 (s) (3H)
1″	26.1	4.19 (d, 6.8) (2H)
2″	124.8	5.37 (td, 6.8, 1.5) (1H)
3″	132.0	-
4″	25.9	1.67 (s) (3H)
5″	18.0	1.84 (s) (3H)

**Linolenoylumbilicaxanthoside B**

C_54_H_74_O_17_ (994.49260)

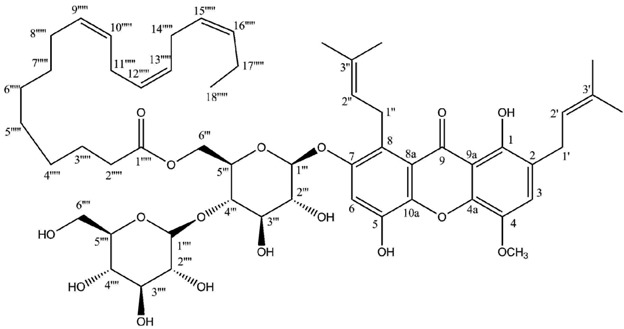


Pale yellow needles, mp 133 °C

UV λ_max_ (MeOH) (log ε): 355 (4.06), 319 (4.01), 271 (3.96), 244 (4.08)

IR ν_max_ (KBr): 3350, 2950, 2900, 1640 cm^−1^

Sources: *Umbilicaria proboscidea* [94].

**Table:** 

	Linolenoylumbilicaxanthoside B (CDCl_3_)	
Position	δ_C_	δ_H_ (*J* in Hz)
1	151.0	-
1–OH	-	13.30 (s) (1H)
2	117.8	-
3	119.4	6.47 (s) (1H)
4	143.2	-
4–OCH_3_	56.3	3.75 (s) (3H)
4a	141.3	-
5	147.1	-
6	103.7	6.42 (s) (1H)
7	153.2	-
8	118.4	-
8a	129.6	-
9	183.1	-
9a	114.6	-
10a	135.7	-
1′	25.4	3.38 (d, 7.2) (2H)
2′	123.8	5.39 (dd, 7.2, 1.2) (1H)
3′	130.9	-
4′	26.2	1.65 (s) (3H)
5′	18.4	1.79 (s) (3H)
1″	26.1	4.19 (d, 6.8) (2H)
2″	124.8	5.37 (dd, 6.8, 1.5) (1H)
3″	132.0	-
4″	25.9	1.67 (s) (3H)
5″	18.0	1.84 (s) (3H)
1‴	99.8	5.05 (d, 8.1) (1H)
2‴	74.2	3.50 (dd, 8.9, 8.1) (1H)
3‴	77.1	3.58 (t, 8.9) (1H)
4‴	76.7	3.05 (dd, 9.3, 8.9) (1H)
5‴	76.1	3.54 (m) (1H)
6‴	65.3	4.51 (dd, 12.1, 2.3) (1H)
		4.03 (dd, 12.1, 5.2) (1H)
1’’’’	104.1	5.15 (d, 8.0) (1H)
2’’’’	73.8	3.52 (dd, 9.0, 8.0) (1H)
3’’’’	76.5	3.61 (t, 9.0) (1H)
4’’’’	69.7	3.41 (t, 9.0) (1H)
5’’’’	76.9	3.45 (m) (1H)
6’’’’	62.6	3.93 (dd, 12.1, 2.2) (1H)
		3.72 (dd, 12.1, 5.2) (1H)
1’’’’’	172.0	-
2’’’’’	34.6	2.25 (m) (2H)
3’’’’’	25.0	1.68 (m) (2H)
4’’’’'	29.2	1.29 (m) (6H)
5’’’’’	29.3	1.29 (m) (6H)
6’’’’’	29.1	1.29 (m) (6H)
7’’’’’	30.3	1.33 (m) (2H)
8’’’’’	27.3	1.96 (m) (2H)
9’’’’’	131.8	5.37 (m) (1H)
10’’’’’	131.7	5.42 (m) (1H)
11’’’’’	25.7	2.68 (m) (2H)
12’’’’’	128.3	5.35 (m) (2H)
13’’’’’	128.4	5.35 (m) (2H)
14’’’’’	25.6	2.73 (m) (2H)
15’’’’’	127.1	5.43 (m) (1H)
16’’’’’	131.9	5.36 (m) (1H)
17’’’’’	20.6	1.93 (m) (2H)
18’’’’’	14.2	1.06 (t, 6.8) (3H)

**Secalonic acid A**

C_32_H_30_O_14_ (638.16356)

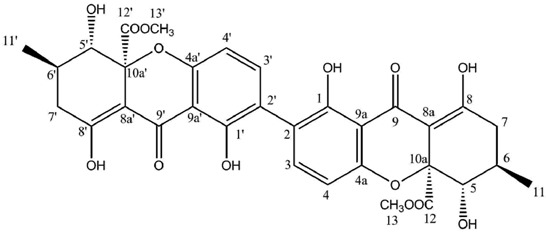


Faint yellow crystals, mp 231–232 °C

${\left[\alpha \right]}_{D}^{26}$ = −64.1 (*c* = 0.1 in CHCl_3_)

UV λ_max_ (MeOH)(log ε): 338 (4.52), 260 (4.12), 228 (4.32) nm

IR ν_max_ (CHCl_3_): 3464, 1726, 1610, 1585, 1566, 1436, 1233, 1067 cm^−1^

Sources: *Diploicia canescens* [117].

Spectral data: [129].

Enantiomeric secalonic acid D: similar physicochemical data except ${\left[\alpha \right]}_{D}^{26}$ = + 64.1 (*c* = 0.14 in CHCl_3_)

**Table:** 

	Secalonic Acid A (CDCl_3_)	
Position	δ_C_	δ_H_ (*J* in Hz)
1, 1′	159.4	-
–OH 1,1′	-	11.75 (s) (2H)
2, 2′	118.2	-
3, 3′	140.2	7.46 (d, 8.5) (2H)
4, 4′	107.6	6.63 (d, 8.5) (2H)
4a, 4a′	158.3	-
5, 5′	76.9	3.93 (dd, 11.3, 1.6) (2H)
–OH 5,5′	-	2.81 (d, 2.2) (2H)
6, 6′	29.2	2.41 (m) (2H)
7, 7′	36.3	2.74 (dd, 19.0, 6.1) (2H)
		2.32 (dd, 19.0, 11.5) (2H)
8, 8′	177.5	-
–OH 8,8′	-	13.78 (s) (2H)
8a, 8a′	101.5	-
9, 9′	187.2	-
9a, 9a′	106.9	-
10a, 10a′	84.7	-
11, 11′	18.0	1.17 (d, 6.4) (6H)
12, 12′	170.3	-
13, 13′	53.3	3.73 (s) (6H)

**Secalonic acid B**

C_32_H_30_O_14_ (638.16356)

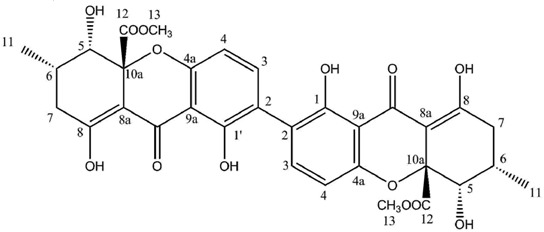


Faint yellow crystals, mp 231–232 °C

${\left[\alpha \right]}_{D}^{20}$ = + 133.7 (*c* = 0.38 in CHCl_3_)

UV λ_max_ (MeOH)(log Ɛ): 338 (4.52), 260 (4.12), 228 (4.32) nm

IR ν_max_ (CHCl_3_): 3582, 3014, 1747, 1611, 1589, 1214, 1058, 796, 726 cm^−1^

Sources: *Diploicia canescens* [117].

Spectral data: [47]

**Table:** 

	Secalonic acid B (CDCl_3_)	
Position	δ_C_	δ_H_ (*J* in Hz)
1, 1′	159.4	-
–OH 1,1′	-	11.85 (s) (2H)
2, 2′	118.7	-
3, 3′	139.7	7.42 (d, 8.5) (2H)
4, 4′	107.5	6.57 (d, 8.5) (2H)
4a, 4a′	157.2	-
5, 5′	71.4	4.12 (d, 1.4) (2H)
–OH 5,5′	-	2.57 (s) (2H)
6, 6′	28.5	2.12 (m) (2H)
7, 7′	32.6	7, 7′ α 2.52 (dd, 19.0, 11.5) (2H)
		7, 7′ β 2.41 (dd, 19.0, 6.1) (2H)
8, 8′	179.8	-
–OH 8,8′	-	13.96 (s) (2H)
8a, 8a′	99.9	-
9, 9′	187.7	-
9a, 9a′	107.0	-
10a, 10a′	84.8	-
11, 11′	17.5	1.18 (d, 6.8) (6H)
12, 12′	171.2	-
13, 13′	53.4	3.72 (s) (6H)

## 7. Conclusions

Even though lichens offer the widest diversity of compounds in the fungal realm, the bioactivities of their secondary metabolites remain under-investigated in regards to any other fungi [130], especially xanthones, despite being widely considered as a promising class of compounds exerting pleiotropic pharmacological activities. Xanthones might have originally suffered from their ubiquitous distribution compared to metabolites restricted to the lichenized condition (e.g., depsides, depsidones, depsones…). However, as end products of a distinct biosynthetic pathway to that of higher plants, lichen xanthones display unique substitution patterns which enrich the diversity of xanthones rather than produce cross-phyletic structures. Furthermore, recently discovered lichen xanthones extend their diversity through unexpected structural features (e.g., prenylations, glycosylations, unprecedented substitution patterns…) and, as a consequence, less than 10% are known from non-lichen sources. In addition, their structural elucidation may be misleading for the spectroscopists, with the presence of multiple possible isomers. Innovative analytical approaches both alleviate and shorten the dereplication holdup while recent advances in spectroscopy ensure reliable structural assignments. Analytical aspects no longer hamper the study of these valuable metabolites.
